# Association of hypothyroidism during pregnancy with preconception and early pregnancy exposure to ambient particulate matter

**DOI:** 10.1007/s11356-023-28683-7

**Published:** 2023-07-12

**Authors:** Qi Sun, Yuanmei Chen, Fang Ye, Jing Liu, Die Liu, Bo Ao, Qin Hui, Qi Zhang

**Affiliations:** National Center for Respiratory Medicine; State Key Laboratory of Respiratory Health and Multimorbidity; National Clinical Research Center for Respiratory Diseases; Institute of Respiratory Medicine, Chinese Academy of Medical Sciences; Department of Pediatrics, China-Japan Friendship Hospital, Beijing, China

**Keywords:** Hypothyroidism, Ambient particulate matter, Risk factor, Pregnancy, Air pollution, Preconception

## Abstract

**Background:**

Limited research has been conducted on the association between preconception exposure to ambient particulate matter (PM) and hypothyroidism. This study aimed to investigate the relationship between preconception PM exposure and hypothyroidism.

**Methods:**

A retrospective case–control study at China-Japan Friendship Hospital was performed. Fine particulate matter (PM_2.5_) and inhalable particulate matter (PM_10_) were obtained from the China High Air Pollution Dataset. Buffer analysis methods were used to calculate the exposure of pregnant women to PM in a circular area of 250, 500, and 750 m in diameter at preconception and in early pregnancy. Logistic regression models were used to assess the relationship between PM and hypothyroidism. Odd ratios (ORs) and 95% confidence intervals (CIs) were used to evaluate the effect of PM on the risk of hypothyroidism.

**Results:**

A total of 3,180 participants were studied, and they comprised 795 hypothyroid patients and 2,385 matched controls. The mean age was 31.01 years (standard deviation: 3.66) in the control group and 31.16 years (standard deviation: 3.71) in the case group. Logistic regression analysis showed that exposure to PM_2.5_ and PM_10_ in the 60-day period before the last menstrual period month (LMPM), 30-day period before the LMPM, and LMP, across all distance buffers, was associated with an increased risk of hypothyroidism (all *P* < 0.05). The most pronounced effect was observed during the LMPM, with PM_2.5_ (OR: 1.137, 95% CI: 1.096–1.180) and PM_10_ (OR: 1.098, 95% CI: 1.067–1.130) in the 250-m buffer. Subgroup analysis in the Changping District yielded consistent results with the main analysis.

**Conclusion:**

Our study shows that preconception PM_2.5_ and PM_10_ exposure increases the risk of hypothyroidism during pregnancy.

## Introduction

Hypothyroidism is a disease characterized by a reduced systemic metabolism owing to a reduction in the synthesis and secretion of thyroid hormones or a decline in the effects of thyroid hormones (Chaker et al. 2022). Hypothyroidism is an important public health problem, and studies have shown that the prevalence of hypothyroidism during pregnancy is approximately 2% in areas with adequate iodine. (Korevaar et al. 2017; Medici et al. 2015). Hypothyroidism during pregnancy has long-term or short-term adverse effects on maternal and fetal outcomes. Hypothyroidism in pregnancy is an important risk factor for adverse pregnancy outcomes, such as preterm birth (Korevaar et al. 2019), miscarriage (Zhang et al. 2017), and pediatric endocrine morbidity in the offspring (Eshkoli et al. 2019), and is a major cause of critical illnesses, such as preeclampsia in pregnancy (Su et al. 2021). Although hypothyroidism is not a direct cause of maternal death, it is still a risk factor for other diseases. Therefore, health education and prevention for pregnant women may reduce the risk of developing hypothyroidism and related complications.

In recent years, the association between diseases and air pollution has gained attention (Bai et al. 2020; Dehghani et al. 2020). The main air pollutants involved are ambient particulate matter (PM), ozone (O_3_), nitrogen dioxide (NO_2_), carbon monoxide (CO), and other trace organic substances and volatile organic compounds, among which PM has the greatest effect on human health (Dehghani et al. 2020; Orellano et al. 2020). Pregnant women represent a unique and vulnerable population when environmental stressors are present. During pregnancy, women undergo various physiological and hormonal changes, which can affect their susceptibility to the effects of environmental stressors (Soma-Pillay et al. 2016; Wang et al. 2021). Maternal exposure to PM can potentially affect the health and well-being of the mother and the developing fetus. Exposure to PM during pregnancy is associated with adverse pregnancy outcomes, such as gestational diabetes (Zhang et al. 2020), fetal growth restriction (Zhou et al. 2022b), and hypertensive disorders in pregnancy (Cao et al. 2021). Investigating the association between PM and thyroid function specifically in pregnant women is important because thyroid hormones play a crucial role in fetal brain development and overall maternal health (Huget‐Penner and Feig 2020). However, few studies have been conducted on air pollutants and hypothyroidism during pregnancy.

The pathogenesis of hypothyroidism in pregnancy is unclear, and may involve genetic factors, lifestyle, dietary, and environmental factors (Dehghani et al. 2022; Maraka et al. 2016). In recent years, there has been increasing concern regarding the potential adverse effects of PM exposure during pregnancy on thyroid function in pregnant women. A study of four European cohorts and one United States cohort showed that fine particulate matter (PM_2.5_) exposure during the first trimester of pregnancy was associated with mild thyroid dysfunction throughout pregnancy (Ghassabian et al. 2019). Studies have shown that maternal free thyroxine (FT4) concentrations are inversely associated with maternal exposure to PM_2.5_ (Wang et al. 2019), but not associated with inhalable particulate matter (PM10) (Zhang et al. 2022). Research suggests that PM_2.5_ may interfere with thyroid function in pregnant women during the first trimester (Zhao et al. 2019).

These studies suggest an association between PM_2.5_ and PM_10_ exposure and thyroid function in pregnant women, However, to the best of our knowledge, no studies have investigated the association between preconception exposure to ambient PM and hypothyroidism during pregnancy. Moreover, evaluations of PM in previous studies were based on city- or county-level atmospheric stations, which may not have accurately estimated the exposure of pregnant women to PM throughout pregnancy. Therefore, the association between preconception exposure to PM and the risk of hypothyroidism during pregnancy and whether there any temporal variations in this association during different time windows need to be determined. To address these issues, this study aimed to (1) investigate the relationship between preconception and early pregnancy PM exposure and the risk of hypothyroidism during pregnancy using a high-precision 1 × 1-km gridded China High Air Pollutant (CHAP) dataset (Wei et al. 2021a); and (2) to examine potential temporal variations in the association between preconception PM exposure and hypothyroidism during different time windows, comprising the 30-day period before the last menstrual period month (LMPM), the 60-day period before the LMPM, and the 90-day period before the LMPM. The findings of our study will contribute to the existing knowledge on the potential health risks associated with PM exposure during pregnancy, providing important insight into the prevention and management of maternal health risks related to PM exposure.

## Methods

### Participants and study design

**Fig. 1:**
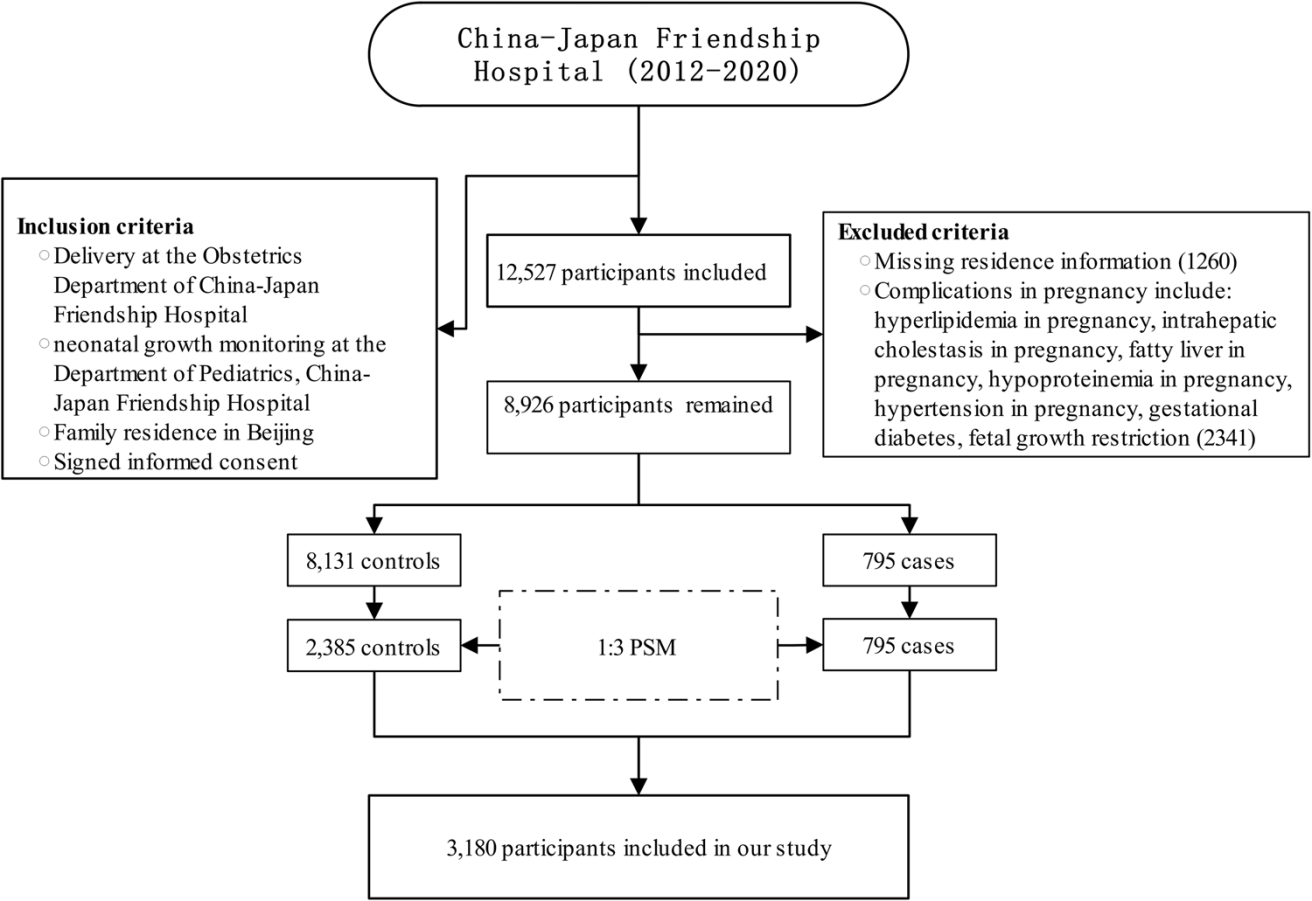
Flowchart of this study. PSM: propensity score matching

This was a case–control study conducted at China-Japan Friendship Hospital. The study population consisted of pregnant women who gave birth at this hospital between 2012 and 2020. The inclusion criteria for the study subjects were as follows: 1) delivery at the Obstetrics Department of China-Japan Friendship Hospital; 2) neonatal growth monitoring was performed at the Pediatrics Department of China-Japan Friendship Hospital after birth, 3) residence in Beijing; and 4) signed informed consent. The exclusion criteria for the study population were as follows: 1) missing residence information; and 2) the women had complications of pregnancy, such as hyperlipidemia, intrahepatic cholestasis, fatty liver, hypoproteinemia, hypertension, diabetes mellitus, and fetal growth restriction. The controls in this study were matched by propensity score matching at a ratio of 1:3 according to age, ethnicity, gravidity, and parity. The workflow of the study is shown in Fig. 1. The study protocol was approved by the Ethical Review Board of China-Japan Friendship Hospital (No: 2022-KY-006–1).

### Assessment of hypothyroidism

Hypothyroidism was diagnosed on the basis of assessment of thyroid-stimulating hormone (TSH) and FT4 concentrations. The diagnostic criteria of hypothyroidism were TSH concentrations > 4 mU/L and FT4 concentrations below the lower limit of the reference range (0.93–1.70 ng/dL) (Ross 2013). Before being enrolled in the study, all participants underwent a minimum of one thyroid function test, which was conducted before the 12th week of pregnancy. Subsequently, participants who were diagnosed with hypothyroidism were classified and assigned codes in accordance with the tenth revision of the International Classification of Diseases (ICD-10) (DiSantostefano 2009).

### Assessment of air pollution

The 1 × 1-km gridded CHAP monthly dataset (available at https://doi.org/10.5281/zenodo.3753614) was used to measure short-term pollutant exposure. In brief, the CHAP dataset is a long-term, full-coverage, high-resolution, high-quality, and ground-gridded source of air pollutant data. The Space–Time Extra-Trees model was used in the CHAP dataset to estimate PM concentrations on the basis of long-term and high spatial resolution aerosol optical depths generated by the Moderate Resolution Imaging Spectroradiometer Multi-Angle Implementation of Atmospheric Correction algorithm (Wei et al. 2021a, b). The Space–Time Extra-Trees model uses several basic information and variables, such as meteorological data (e.g., temperature, humidity, and wind speed), land use/land cover information, and satellite-derived aerosol optical depths, in its training process. These variables were selected on the basis of their known associations with PM concentrations and their availability in the study area. The model’s performance was assessed by using cross-validation and validation against ground-based PM measurements. Strong predictive capabilities with high accuracies were observed in the model. The cross-validation coefficient of determination (CV-R^2^) for PM_2.5_ ranged from 0.86 to 0.90, with an average root mean square error ranging from 10.0 to 18.4 μg/m^3^. With regard to PM_10_, the dataset showed a high quality, with a cross-validation coefficient of determination ranging from 0.83 to 0.87 and a small root mean square error ranging from 19.7 to 28.4 μg/m^3^. The CHAP dataset provided daily and monthly concentrations of PM_1_, PM_2.5_, PM_10_, O_3_, NO_2_, SO_2_, and CO, and covered all regions of China from 2000 to 2022 with no missing data.

In this study, the participants’ air pollutant exposure concentrations of ambient PM were estimated through a two-step strategy. In the first step, we geocoded the residence address information of the participants using the application program interface provided by the open platform of Baidu Maps in China (https://lbsyun.baidu.com/). In the second step, we estimated the average exposure concentrations of air pollutants for participants in 250-, 500-, and 750-m diameter circular buffer zones based on each residential location.

### Exposure time window

**Fig. 2:**
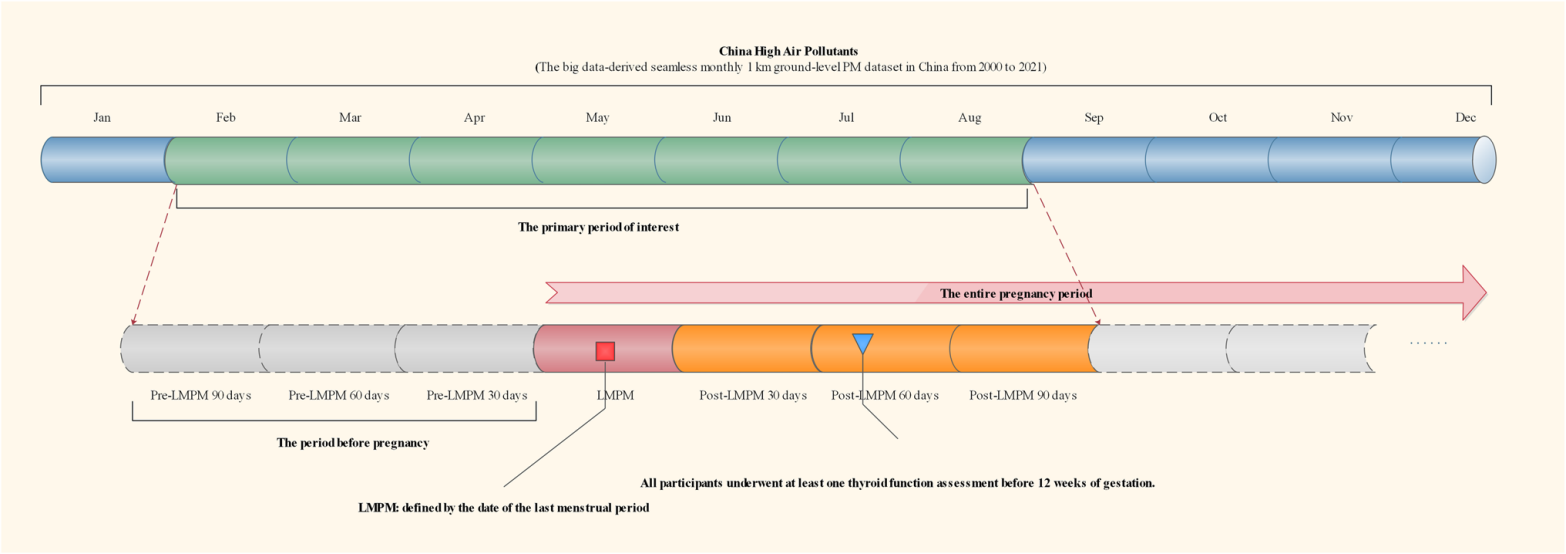
Exposure time window of the study

To assess the potential effects of pollutant exposure on hypothyroidism during pregnancy, we defined an exposure window according to the last menstrual period month (LMPM) of pregnant women. The exposure window encompassed the preconception and post-LMPM periods. Preconception exposure was defined as 30, 60, and 90 days preceding the LMPM. Additionally, we assessed pollutant exposure at 30, 60, and 90 days following the LMPM. This post-LMPM exposure assessment enabled us to examine the potential effect of early gestational pollutant exposure on hypothyroidism (Fig. 2).

### Statistical analyses

Continuous variables are expressed as the mean (standard deviation), and categorical data are expressed as the number (percentage). Differences in continuous variables between groups were compared using the t-test or Wilcoxon test. Differences in categorical variables between groups were compared using the chi-square test or Fisher’s test.

Propensity score matching was used to match controls using age, ethnicity, gravidity, and parity at a 1:3 ratio. Logistic regression models were used to evaluate the association between airborne PM and hypothyroidism during pregnancy. All logistic regression models were adjusted for age, the season of the last menstruation, and pregnancy and delivery history. Odds ratios (ORs) and 95% confidence intervals (CIs) were used to assess the effect of airborne PM exposure on thyroid disease in pregnancy.

All statistical analyses were performed using R (version 4.1.0, available from: https://www.r-project.org/), and the “MatchIt” package of R was used to perform propensity score matching. The R package “pheatmap” was used to visualize the relationship between ambient PM and hypothyroidism during pregnancy. Quantum Geographical Information System (QGIS, version: 3.26.3) software (available from: http://qgis.osgeo.org) was used to perform spatial analysis and mapping. All P values are two-sided, and significance was set at *P* < 0.05.

## Results

**Fig. 3:**
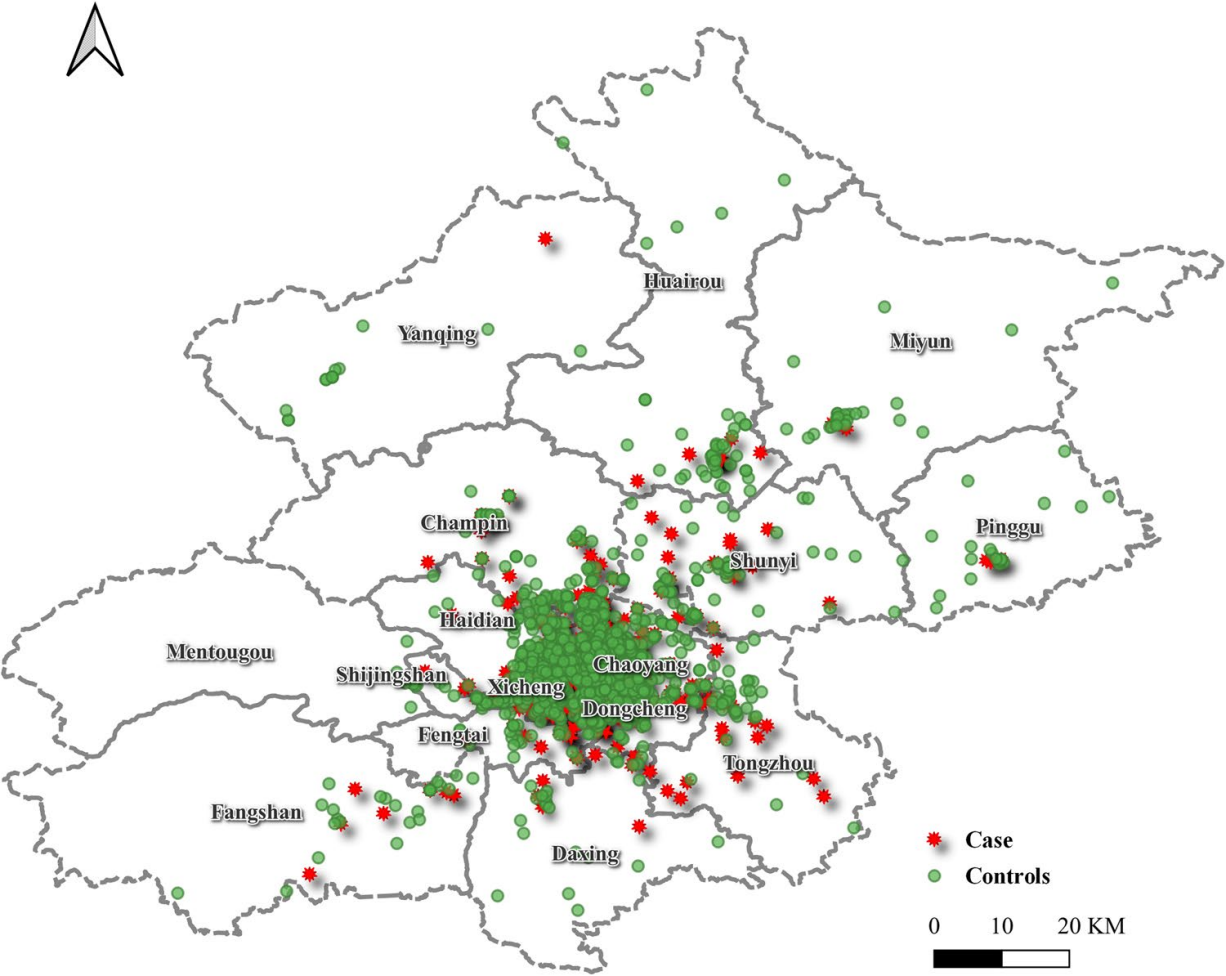
Geographical distribution of pregnant women included in the study. The controls are shown as green dots and the cases are shown as red stars

A total of 3,180 women were enrolled in the study, of whom 795 were hypothyroid patients and 2,385 were matched controls. There were no significant differences in ethnicity, age, gravidity, multiparity, newborn sex, birth length, birth weight, or days of gestation between the two groups (Table 1). The proportion of the LMPM in winter in the case group was higher than that in control group (*P* < 0.05). The geographical distribution of the study participants is shown in Fig. 3. Cases and controls were distributed evenly in Beijing, and most of the study participants were from the Chaoyang District.

**Table 1:** Comparison of characteristics of participants in the study between the case and control groups

		Control group (*n* = 2385)	Case group (*n* = 795)	*P*
Han Chinese (%)	No	116 (4.9)	40 (5.0)	0.924
	Yes	2269 (95.1)	755 (95.0)	
Age (years)		31.01 (3.66)	31.16 (3.71)	0.320
Multipara	No	1926 (80.8)	657 (82.6)	0.260
	Yes	459 (19.2)	138 (17.4)	
Gravidity (times)	1	1476 (61.9)	474 (59.6)	0.472
	2	568 (23.8)	196 (24.7)	
	> 2	341 (14.3)	125 (15.7)	
Neonatal sex (%)	Male	1228 (51.5)	424 (53.3)	0.389
	Female	1157 (48.5)	371 (46.7)	
Neonatal length (cm)		50.58 (2.36)	50.48 (2.38)	0.296
Birth weight (g)		3297.18 (486.74)	3285.73 (482.09)	0.565
LMPM season (%)	Spring	430 (18.0)	209 (26.3)	< 0.001
	Summer	755 (31.7)	195 (24.5)	
	Autumn	763 (32.0)	175 (22.0)	
	Winter	437 (18.3)	216 (27.2)	
Days of gestation (days)		276.22 (12.07)	276.58 (12.76)	0.466
TSH (uIU/mL)		1.72 (0.69)	5.22 (0.75)	< 0.001
FT4 (ng/dL)		1.32 (0.13)	0.71 (0.15)	< 0.001

LMPM: last menstrual period month; TSH: thyroid-stimulating hormone; FT4: free thyroxine; n: number of participants

The differences in preconception and early pregnancy exposure to PM_2.5_ and PM_10_ between the two groups of pregnant women at 250-, 500-, and 750-m buffer distances are shown in Table 2. The average concentration of PM_10_ was significantly higher in the case group compared to the control group at 30 days pre-LMPM, the LMPM, and 30 days post-LMPM for all three buffer distances (*P* < 0.05). Furthermore, the case group exhibited significantly higher average PM2.5 concentrations compared to the control group at 60 days pre-LMPM, 30 days pre-LMPM, the LMPM, and 30 days post-LMPM for all three buffer distances (*P* < 0.05).

**Table 2:** Pregnant women exposed to PM_2.5_ at 250, 500, and 750 m buffer distances at preconception and in early pregnancy

	PM_2.5_			PM_10_		
	Control group	Case group	P	Control group	Case group	P
250 m buffer						
Pre-LMPM 90 days	69.63 (29.67)	68.86 (28.37)	0.526	110.03 (36.15)	106.67 (35.22)	0.023
Pre-LMPM 60 days	67.09 (27.39)	70.10 (30.66)	0.009*	107.35 (35.32)	109.15 (37.75)	0.220
Pre-LMPM 30 days	62.78 (23.72)	67.47 (29.50)	< 0.001*	102.28 (31.41)	106.10 (36.13)	0.004
LMPM	58.37 (19.14)	67.04 (28.69)	< 0.001*	95.79 (26.78)	105.50 (35.66)	< 0.001
Post-LMPM 30 days	62.19 (23.24)	66.13 (26.74)	< 0.001*	99.29 (30.71)	104.45 (33.51)	< 0.001
Post-LMPM 60 days	65.04 (27.21)	66.28 (28.63)	0.275	101.91 (34.32)	104.63 (35.72)	0.055
Post-LMPM 90 days	64.45 (28.05)	65.23 (27.53)	0.499	100.92 (34.65)	103.27 (34.70)	0.098
500 m buffer						
Pre-LMPM 90 days	69.54 (29.60)	68.81 (28.30)	0.545	110.01 (36.09)	106.67 (35.13)	0.023
Pre-LMPM 60 days	67.00 (27.33)	70.03 (30.59)	0.009*	107.34 (35.27)	109.15 (37.66)	0.220
Pre-LMPM 30 days	62.70 (23.70)	67.38 (29.37)	< 0.001*	102.28 (31.42)	106.09 (36.01)	0.004*
LMPM	58.30 (19.09)	66.95 (28.57)	< 0.001*	95.79 (26.76)	105.43 (35.47)	< 0.001*
Post-LMPM 30 days	62.09 (23.18)	66.02 (26.63)	< 0.001*	99.29 (30.67)	104.40 (33.34)	< 0.001*
Post-LMPM 60 days	64.95 (27.14)	66.18 (28.55)	0.274	101.92 (34.29)	104.65 (35.63)	0.054
Post-LMPM 90 days	64.37 (27.97)	65.14 (27.48)	0.502	100.93 (34.59)	103.29 (34.68)	0.097
750 m buffer						
Pre-LMPM 90 days	69.44 (29.51)	68.73 (28.23)	0.550	110.09 (36.06)	106.75 (35.06)	0.023
Pre-LMPM 60 days	66.90 (27.27)	69.95 (30.53)	0.008*	107.42 (35.23)	109.22 (37.60)	0.221
Pre-LMPM 30 days	62.60 (23.65)	67.28 (29.23)	< 0.001*	102.36 (31.44)	106.14 (35.90)	0.005*
LMPM	58.23 (19.03)	66.84 (28.42)	< 0.001*	95.89 (26.74)	105.45 (35.30)	< 0.001*
Post-LMPM 30 days	62.00 (23.12)	65.90 (26.51)	< 0.001*	99.39 (30.64)	104.44 (33.23)	< 0.001*
Post-LMPM 60 days	64.85 (27.06)	66.08 (28.45)	0.275	102.03 (34.26)	104.73 (35.52)	0.057
Post-LMPM 90 days	64.28 (27.88)	65.02 (27.40)	0.518	101.02 (34.52)	103.33 (34.59)	0.103

LMPM: last menstrual period month. the units of ambient particulate matter were μg/m^3^. *P < 0.05

**Fig. 4:**
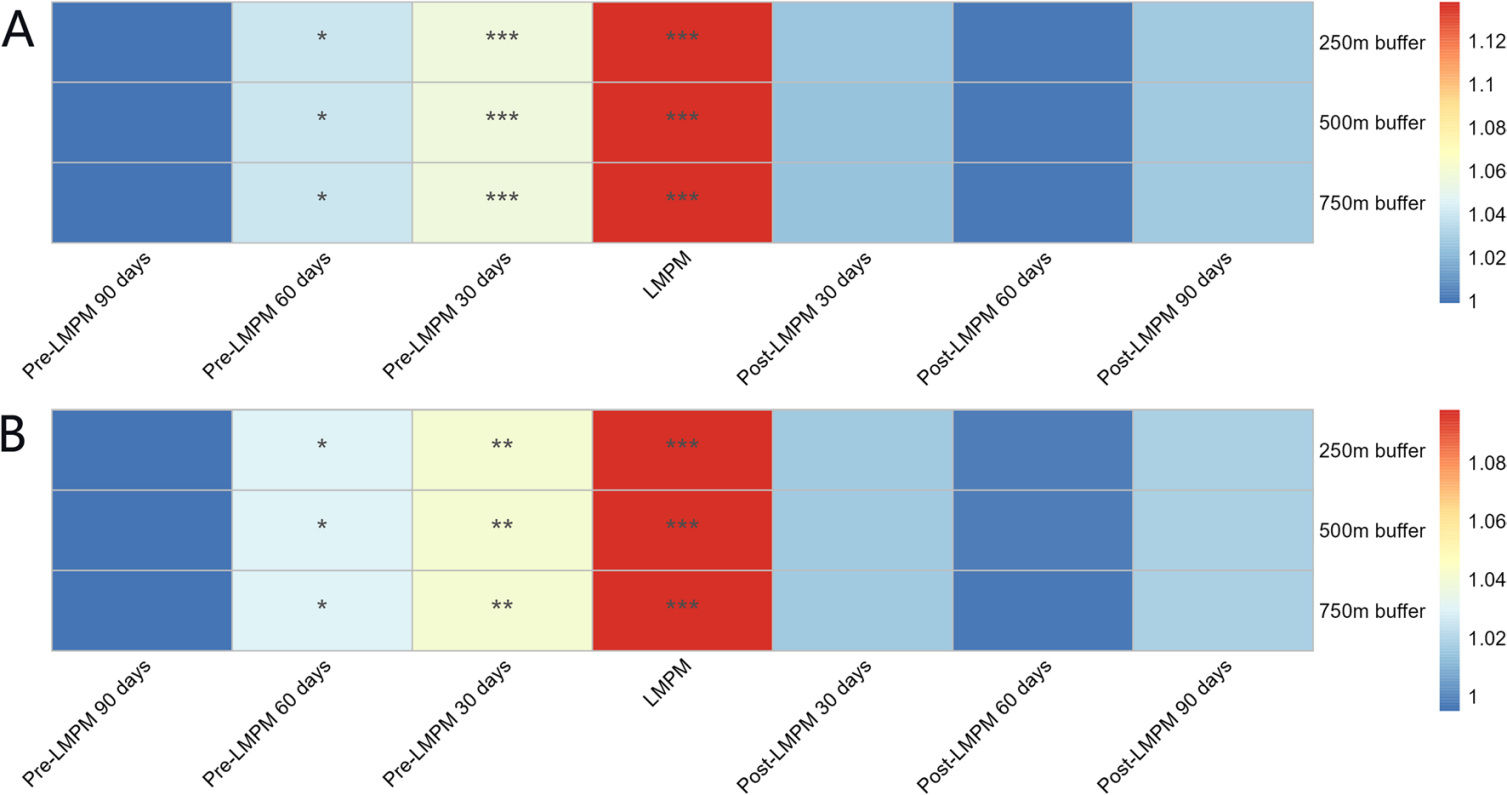
Heat map of OR values for logistic regression of ambient particulate matter and hypothyroidism. LMP: last menstrual period. A: ORs of logistic regression of PM_2.5_ and hypothyroidism; B: ORs of logistic regression of PM_10_ and hypothyroidism. OR values represent the increased risk of hypothyroidism per 10 µg/m.^3^ of ambient particulate matter exposure. *P < 0.05; **P < 0.01 ***P < 0.001

Table 3 shows the logistic regression results of the association between hypothyroidism during pregnancy and air pollution, such as PM_2.5_ and PM_10_. Significant differences in the ORs of PM_2.5_ and PM_10_ were found at 60 days pre-LMPM, 30 days pre-LMPM 30 days, and the LMPM for all three distance buffers. No significant differences were found in the ORs of PM_2.5_ or PM_10_ during the post-LMPM periods for any distance buffer. Furthermore, the strongest significant difference in PM_2.5_ and PM_10_ was observed during the LMPM period across all distance buffers, with ORs of 1.137 (95% CI: 1.096–1.180) and 1.098 (95% CI: 1.067–1.130), respectively. The ORs at 30 days pre-LMPM were also significantly different from 1 for all distance buffers in both models, indicating that this time could also be important for the studied association. In addition, the ORs of PM_10_ and PM_2.5_ at 90 days pre-LMPM were not significantly different from 1 for all distance buffers. Overall, these results suggested that the LMPM period and the 30-day pre-LMPM period were the most important time periods for the association between hypothyroidism during pregnancy and air pollution. The heat map of OR values for logistic regression of ambient PM and hypothyroidism in pregnancy is shown in Fig. 4.

**Table 3:** Relationships between hypothyroidism during pregnancy and exposure to PM_2.5_ and PM_10_

	PM_2.5_		PM_10_	
	OR (95% CI)	P	OR (95% CI)	P
250 m buffer				
Pre-LMPM 90 days	0.999(0.968–1.030)	0.937	0.995(0.970–1.021)	0.716
Pre-LMPM 60 days	1.040(1.008–1.072)	0.012*	1.030(1.004–1.055)	0.021*
Pre-LMPM 30 days	1.056(1.023–1.090)	< 0.001*	1.041(1.013–1.070)	0.004*
LMPM	1.137(1.096–1.180)	< 0.001*	1.098(1.067–1.130)	< 0.001*
Post-LMPM 30 days	1.026(0.991–1.062)	0.150	1.016(0.989–1.044)	0.241
Post-LMPM 60 days	1.001(0.971–1.033)	0.930	0.998(0.973–1.023)	0.868
Post-LMPM 90 days	1.028(0.994–1.062)	0.100	1.018(0.992–1.046)	0.179
500 m buffer				
Pre-LMPM 90 days	0.999(0.969–1.031)	0.966	0.996(0.971–1.021)	0.734
Pre-LMPM 60 days	1.040(1.009–1.072)	0.012*	1.030(1.005–1.056)	0.020*
Pre-LMPM 30 days	1.056(1.023–1.090)	< 0.001*	1.041(1.013–1.070)	0.004*
LMPM	1.138(1.097–1.181)	< 0.001*	1.098(1.066–1.130)	< 0.001*
Post-LMPM 30 days	1.025(0.990–1.061)	0.157	1.016(0.989–1.044)	0.254
Post-LMPM 60 days	1.001(0.970–1.033)	0.937	0.998(0.973–1.023)	0.864
Post-LMPM 90 days	1.028(0.994–1.063)	0.102	1.018(0.991–1.046)	0.181
750 m buffer				
Pre-LMPM 90 days	1.000(0.969–1.031)	0.978	0.996(0.971–1.021)	0.737
Pre-LMPM 60 days	1.040(1.009–1.073)	0.011*	1.030(1.005–1.056)	0.020*
Pre-LMPM 30 days	1.056(1.023–1.090)	< 0.001*	1.041(1.013–1.070)	0.004*
LMPM	1.138(1.097–1.181)	< 0.001*	1.098(1.066–1.130)	< 0.001*
Post-LMPM 30 days	1.025(0.990–1.061)	0.165	1.016(0.988–1.044)	0.270
Post-LMPM 60 days	1.001(0.970–1.033)	0.950	0.997(0.972–1.023)	0.843
Post-LMPM 90 days	1.028(0.994–1.062)	0.108	1.018(0.991–1.045)	0.191

LMPM: last menstrual period month.; OR (95% CI): odds ratio (95% confidence interval); OR values represent the increased risk of hypothyroidism per 10 µg/m^3^ of ambient particulate matter exposure. *P < 0.05. All logistic regression models were adjusted for age, the season of the last menstruation, and pregnancy and delivery history

Table 4 shows the subgroup analysis of the relationship between hypothyroidism during pregnancy and air pollution in the population of Changping District. The results of the subgroup analysis were consistent with the results of the main analysis. Significant differences in the ORs for PM_2.5_ and PM_10_ at 60 days pre-LMPM, 30 days pre-LMPM 30 days, and the LMPM were found at all three distance buffers. No significant differences were found in the ORs during the post-LMPM periods for any distance buffer. The strongest significant difference in PM_2.5_was observed during the LMPM at the 750-m buffer (OR: 1.140, 95% CI: 1.088–1.196). The strongest significant difference in PM_10_ was observed during the LMPM at the 250-m buffer (OR: 1.100, 95% CI: 1.060–1.141).

**Table 4:** Relationships between hypothyroidism during pregnancy and exposure to PM_2.5_ and PM_10_ in Changping District

	PM_2.5_		PM_10_	
	OR	P	OR	P
250 m buffer				
Pre-LMPM 90 days	1.012(0.973–1.052)	0.561	1.005(0.973–1.037)	0.771
Pre-LMPM 60 days	1.052(1.012–1.093)	0.009*	1.042(1.009–1.075)	0.011*
Pre-LMPM 30 days	1.070(1.029–1.113)	< 0.001*	1.053(1.019–1.090)	0.002*
LMPM	1.139(1.088–1.194)	< 0.001*	1.100(1.060–1.141)	< 0.001*
Post-LMPM 30 days	1.038(0.995–1.083)	0.086	1.025(0.990–1.060)	0.160
Post-LMPM 60 days	1.008(0.971–1.047)	0.666	1.006(0.975–1.038)	0.702
Post-LMPM 90 days	1.038(0.996–1.082)	0.072	1.030(0.997–1.065)	0.077
500 m buffer				
Pre-LMPM 90 days	1.012(0.973–1.053)	0.538	1.005(0.973–1.038)	0.769
Pre-LMPM 60 days	1.052(1.013–1.093)	0.009*	1.042(1.009–1.075)	0.012*
Pre-LMPM 30 days	1.070(1.029–1.113)	< 0.001*	1.054(1.019–1.090)	0.002*
LMPM	1.140(1.088–1.195)	< 0.001*	1.099(1.059–1.141)	< 0.001*
Post-LMPM 30 days	1.038(0.994–1.083)	0.090	1.025(0.990–1.060)	0.164
Post-LMPM 60 days	1.008(0.970–1.047)	0.670	1.006(0.975–1.038)	0.702
Post-LMPM 90 days	1.038(0.996–1.082)	0.072	1.030(0.996–1.065)	0.080
750 m buffer				
Pre-LMPM 90 days	1.013(0.973–1.053)	0.532	1.005(0.973–1.038)	0.772
Pre-LMPM 60 days	1.053(1.013–1.094)	0.009*	1.042(1.009–1.075)	0.012*
Pre-LMPM 30 days	1.071(1.029–1.114)	< 0.001*	1.053(1.018–1.089)	0.003*
LMPM	1.140(1.088–1.196)	< 0.001*	1.099(1.059–1.140)	< 0.001*
Post-LMPM 30 days	1.037(0.994–1.083)	0.094	1.024(0.990–1.060)	0.171
Post-LMPM 60 days	1.008(0.970–1.047)	0.685	1.006(0.974–1.038)	0.722
Post-LMPM 90 days	1.038(0.996–1.082)	0.078	1.030(0.996–1.065)	0.085

LMPM: last menstrual period month.; OR (95% CI): odds ratio (95% confidence interval). OR values represent the increased risk of hypothyroidism per 10 µg/m^3^ of ambient particulate matter exposure. *P < 0.05. All logistic regression models were adjusted for age, the season of the last menstruation, and pregnancy and delivery history

## Discussion

This study investigated the association between preconception and early pregnancy exposure to PM and the risk of hypothyroidism during pregnancy. Using a retrospective case–control study design, in combination with the highest resolution exposure data available in the Chinese region, we found that PM at 60 days before conception and at the month of conception was a risk factor for hypothyroidism during pregnancy. The subgroup analysis and buffering zone analysis with different ranges further confirmed the stability of our results.

Previous studies have associated long-term exposure to air pollutants with diseases, such as cardiovascular disease, lung disease, and adverse reproductive outcomes (Boogaard et al. 2022; Franklin et al. 2015; Yang et al. 2021; Zhou et al. 2022a). In this study, we found that exposure to PM in the first month of pregnancy was a risk factor for hypothyroidism during pregnancy, which is consistent with previous studies (Ghassabian et al. 2019; Zhang et al. 2022; Zhao et al. 2019). Zhao et al. showed that PM exposure during early and mid-pregnancy was a risk factor for hypothyroidism during pregnancy (Zhao et al. 2019). Furthermore, another study showed an association between early pregnancy exposure to PM_2.5_ and reduced FT4 concentrations, which are an indicator of hypothyroidism (Zhang et al. 2022). A large European population cohort study showed that higher exposure to PM_2.5_ was associated with a higher risk of hypothyroxinemia, but exposure to PM_10_ was not significantly associated with hypothyroxinemia (Ghassabian et al. 2019). The above-mentioned studies established a relationship between ambient air pollution exposure during early pregnancy and hypothyroidism. However, limited research has investigated the association between air pollution exposure during preconception and hypothyroidism.

In the present study, we found that exposure to air pollution during the LMP, and at 30 and 60 days before the conception month was a risk factor for hypothyroidism. The preconception period is a crucial stage in pregnancy. Good preconception care can reduce the incidence of fetal malformations and improve the health of the fetus (Berghella et al. 2010). A growing number of studies have shown associations between preconception air pollution exposure and maternal health and fetal outcomes during pregnancy. One study showed an association between preconception air pollutant exposure and the risk of congenital heart disease in newborns (Vecoli et al. 2016). A meta-analysis of 31 cohort studies showed that exposure to PM_2.5_ and PM_10_ was associated with an increased risk of gestational diabetes, especially at preconception and the first trimester of pregnancy (Liang et al. 2023). A systematic review showed that maternal pre-pregnancy exposure to PM_2.5_ and PM_10_ was associated with child health, including birth defects, preterm birth, low birth weight, and autism (Blanc et al. 2023). Therefore, additional research is required to validate the association between preconception exposure to air pollution and hypothyroidism. Nevertheless, because air pollution exposure is a modifiable risk factor, it should be avoided before conception.

The mechanisms underlying the effect of preconception exposure to environmental PM on hypothyroidism remain poorly understood. Potential mechanisms may involve genetic factors, oxidative stress, inflammation, and metabolic dysregulation. Previous studies have suggested genetic involvement in the association between environmental exposures and disease (Moubarz et al. 2023; Saad-Hussein et al. 2022). Exposure to environmental PM can affect thyroid function by modulating gene expression and signaling pathways associated with the thyroid (Wen et al. 2021). Additionally, individual genetic variations, such as gene polymorphisms, may affect the sensitivity and metabolic capacity toward environmental PM, thereby contributing to individual differences in the effects on thyroid function (Huang et al. 2004). Inflammation is one possible mechanism of PM exposure because environmental PM can enter the body through the respiratory system, leading to an association with inflammatory responses and potential effects on thyroid function (Jiang et al. 2021; Liu et al. 2022). Another potential mechanism is endocrine disruption in which certain components of air pollution, such as polycyclic aromatic hydrocarbons, possess endocrine-disrupting properties that interfere with the normal functioning of the thyroid gland (Darbre 2018; Dehghani et al. 2022). Additionally, oxidative stress is considered another potential mechanism because air pollutants have the ability to generate reactive oxygen species and induce oxidative stress, resulting in damage to thyroid tissue and impairment of thyroid hormone synthesis and function (Chen et al. 2023). Further research is required to gain a deeper understanding of these mechanisms and their interplay to determine the complex relationship between preconception environmental PM exposure and thyroid dysfunction.

In our study, no significant association was found between air pollution exposure from weeks 4 to 18 of pregnancy and hypothyroidism, which is inconsistent with a previous study (Zhang et al. 2022). One possible reason for this outcome is that our participants’ outcomes were defined in the early stages of pregnancy (before 12 weeks’ gestation). Therefore, we cannot ascertain whether PM exposure from weeks 4 to 18 of pregnancy occurs before the onset of hypothyroidism. Consequently, inferring a causal relationship between environmental PM exposure and hypothyroidism is difficult. This issue may be one of the factors contributing to the observed disparity in results between studies. In this study, different buffer distances of 250, 500, and 750 m were used to accurately assess the exposure to air pollutants in pregnant women. The buffer analysis was used to establish buffer zones at various distances to investigate air pollutant concentrations at different ranges, which is a method extensively used in previous studies. (Graafland et al. 2023; Mendrinos et al. 2022). In our study, differences in PM_10_ and PM_2.5_ between the groups were observed at all three buffer zones, which indicates the stability of our results.

Overall, the present study provides considerable insight into the health risks associated with air pollution exposure during preconception and early pregnancy, emphasizing the necessity for effective interventions to mitigate exposure to air pollutants and protect maternal well-being. Nonetheless, we need to acknowledge the inherent limitations to our study. First, our study was conducted at a single center, necessitating further validation and investigation before generalizing the findings to broader populations or domains. Subsequent investigations should use a multicenter, multi-regional study design. Second, the assessment of indoor pollution was not performed when measuring PM exposure. Consequently, future studies should comprehensively examine the association between indoor and outdoor pollution exposure and decreased thyroid function during pregnancy. Third, the sample size in this study was relatively small. To avoid ecological fallacies, the study population was limited to the Beijing area, with a subgroup analysis conducted on the Chaoyang District population, which demonstrated the robustness of the findings. Fourth, the number of positive samples in this study was limited, and propensity score matching was used taking into consideration factors, such as maternal age, ethnicity, the number of pregnancies, and the number of births, to minimize selection bias in control selection. However, certain confounding factors, such as smoking and alcohol consumption habits, were not accounted for in our study. A previous study showed an association between smoking behavior and elevated concentrations of thyroid hormones (Gruppen et al. 2020). Therefore, future studies should consider the effect of these confounding factors on the outcomes.

## Conclusions

This study shows that exposure to PM_2.5_ and PM_10_ during the preconception and early pregnancy periods poses a considerable risk of thyroid disease in pregnancy, particularly on exposure during the month of conception. These results highlight the urgent requirement for comprehensive strategies to address air pollution and protect maternal thyroid health. Policymakers and healthcare professionals should prioritize interventions aimed at reducing air pollution levels and promoting awareness regarding its detrimental effects on pregnancy outcomes. Further research is warranted to determine the underlying mechanisms and long-term implications of air pollution on thyroid health in pregnancy. This study provides important insight that contributes to the growing body of evidence on the association of air pollution and maternal health. This evidence suggests the importance of implementing targeted interventions and policies to mitigate the adverse effects of air pollution on pregnant women.

## Data Availability

The datasets used or analyzed during the current study are available from the corresponding author on reasonable request.
